# Physiological and Physical Profile of Snowboarding: A Preliminary Review

**DOI:** 10.3389/fphys.2018.00770

**Published:** 2018-06-20

**Authors:** Gianluca Vernillo, Cesare Pisoni, Gabriele Thiébat

**Affiliations:** ^1^Department of Biomedical Sciences for Health, University of Milan, Milan, Milan; ^2^Human Performance Laboratory, Faculty of Kinesiology, University of Calgary, Calgary, AB, AB; ^3^Snowboard and Freestyle Sector, Italian Winter Sports Federation, Milan, Milan; ^4^Sports Trauma Centre, IRCCS Institute Orthopedic Galeazzi, Milan, Milan

**Keywords:** olympics, performance, physiological capabilities, test, training, snowboarding, winter sports

## Abstract

The sport of snowboarding has grown in popularity as both a recreational winter activity as well as a prominent Olympic sport. Both forms are comprised of one of three different disciplines within the sport: freestyle, alpine, and snowboard-cross. In recent years, the increased professionalism and substantial growth of snowboarding as a global sport has increasingly attracted the interest of exercise physiologists and sport scientists. Given the small (but growing) number of studies that have been published, the research analyzing the physiological and performance characteristics and requirements of snowboarding remains limited. The absence of such studies signifies a lack of examination into this important but under-explored area of research, which could contribute valuable information to the scientific community and international snowboarding teams. The studies conducted thus far have indicated different requirements of physiological and physical traits dependent upon the specific discipline of snowboarding in question. For example, in order to meet the divers demands of each discipline, athletes must develop various qualities, such as muscular strength and power. This can increase their ability to withstand the high forces and loads on the muscular system during competition, and further decrease their risk of lower limbs injuries. At the same time, the studies acknowledge the potential advantages of aerobic fitness in terms of recovery, to more efficiently sustain the athlete through both competitive and on- and off-snow training sessions. Given the value and breadth of application of these limited studies, further analysis and research could contribute greater knowledge and benefits to the field of snowboarding. Therefore, it is the purpose of this preliminary review to explore the current literature, providing further insight into the physiological and physical demands of snowboarding performance. This preliminary review is intended to stimulate interest among the communities of exercise physiologists, sport scientists and particularly coaches in order to improve our current understanding of snowboarding and its demands as a sport. This preliminary review further seeks to develop protocols and strategies to assess physiological and performance characteristics of snowboarding, monitor athletic performance, provide practical recommendations for training, identify new areas of scientific research, and develop accurate talent identification programs.

## Introduction

Snowboarding in its current form began in the United States in the 1960s, and is now one of the most popular winter sports. In recognition of the trend, the International Olympic Committee officially introduced snowboarding into the Olympic program since Nagano 1998. Despite some decline over the last 10 years, snowboarding remains key among winter sports and is represented by a high number of participants at most alpine resorts worldwide (Bladin et al., 2004).

Traditionally, snowboarding is described as (i) freestyle (SBfs) - a skill-based discipline where athletes perform tricks and jumps either on the slopes or using specially built rails and half pipes; (ii) snowboard-cross (SBx) - where four to six athletes are required to maneuver inside a course characterized by multiple obstacles (e.g., banks and jumps); and (iii) alpine (SBalp) - where two athletes are required to ride simultaneously side-by-side down two parallel courses through gates with tight turns (Vernillo et al., 2016a).

Snowboarding as an athletic sport tests the boundaries of both physical and technical competence. The more we learn regarding the physiological demands placed upon elite snowboarders, the more effectively these qualities can be replicated and improved upon in the athletes. Knowledge of the muscular forces and energy systems involved in snowboarding is important for training prescription, performance enhancement, injury prevention and talent identification. However, for many years snowboarding studies have been limited to the realm of injuries [e.g., (Davidson and Laliotis, 1996; Machold et al., 2000; Langran and Selvaraj, 2002; Bladin et al., 2004; Torjussen and Bahr, 2005; Hasler et al., 2010; Bakken et al., 2011; Steenstrup et al., 2011; Ishimaru et al., 2012; Mahmood and Duggal, 2014; Schmitt and Muser, 2014; Wijdicks et al., 2014)] and biomechanical factors (Wu et al., 2006; Klous et al., 2010). More recently, snowboarding has attracted the interest of exercise physiologists and sport scientists, resulting in a small but growing number of studies being published. However, the extent of this research is based on the few available studies that analyze the physiological and performance requirements of snowboarding, and therefore limited information can be gleaned. We believe this is an important though neglected area of research, which could offer vital information to the scientific community as well as snowboarding teams throughout the globe. This preliminary review explores the current literature to provide insights into the physiological and physical characteristics of snowboarding performance. Our aim is to stimulate exercise physiologists, sport scientists and particularly coaches to improve their understanding of snowboarding demands in order to develop protocols and strategies to better assess physiological and performance characteristics of snowboarding, monitor snowboarders performance, provide practical recommendations for training as well as new areas of scientific research and develop accurate talent identification programs. Anthropometric variables and other factors that may be important in determining snowboarding performance are also discussed. Of note, the terms “snowboarders” and “snowboarding athletes” are frequently and interchangeably used throughout the following discussion, indicating the same population of athletes unless otherwise stated.

## Physiological and Physical Profile of Snowboarding

### Anthropometric Characteristics

Several studies describe the anthropometric variables present in elite snowboarders (**Table 1**). The mean height is between 165.7 cm and 183.4 cm. The average body mass of elite men Italian snowboarders was 76.0 ± 9.7 kg (Vernillo et al., 2016b). This value is similar to that reported in a study of elite Austrian men snowboarders (75.4 ± 9.9 kg) (Platzer et al., 2009). Gathercole et al. (2015) reported that the average body mass of men and women Canadian SBx athletes was ∼86 kg and 64 kg, respectively. Body composition seems to be of similar import. Indeed, the average body fat percentage has been reported to be between 12 and 14% in elite men Italian snowboarders (Vernillo et al., 2016a). Taken together, these data argue for the potential importance of physique (as well as body composition) for snowboarding performance. Indeed, these characteristics may serve to manage the demands arising from fast and responsive turns and changing edges as well as negotiating obstacles. However, studies have incorporated only a small selection of anthropometric variables as part of investigations undertaken with different aims. Therefore, a more comprehensive data set on the anthropometric characteristics of elite snowboarders is missing and its quantification should be further investigated. In doing so, opportunities may arise to better identify anthropometric qualities key to snowboarding performance.

**Table 1:** Maximum oxygen uptake (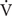
O_2max_) and anthropometric characteristics of snowboarders reported in the literature.

Study (year)	Competitive level	Discipline	Sample size	Height (cm)	Body mass (kg)	Body fat (%)	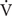 O_2max_ (mLO_2_⋅kg^-1^⋅min^-1^)	Mean aerobic power (W⋅kg^-1^)
Platzer et al., 2009	Elite	–	16 (women)	167 ± 5	59.7 ± 5.3	–	–	range: 3.5–4.7
			21 (men)	177 ± 6	75.4 ± 9.9	–	–	range: 3.8–5.3
								
Gathercole et al., 2015	Elite	SBx	3 (women)	165.7 ± 4.4	64.4 ± 4.5	–	–	–
			4 (men)	183.4 ± 3.8	86.2 ± 3.4	–	–	–
								
Vernillo et al., 2016a	Elite	SBx	10 (men)	181.0 ± 4.9	77.2 ± 9.2	11.9 ± 3.5	51.2 ± 4.5	4.5 ± 0.3
		SBalp	10 (men)	178.4 ± 9.8	78.1 ± 12.1	13.8 ± 3.7	49.7 ± 3.8	4.6 ± 0.5
								
Vernillo et al., 2016b	Elite	SBfs	10 (men)	178.4 ± 7.9	72.8 ± 9.7	–	–	–
		SBx	11 (men)	181.7 ± 5.3	77.5 ± 8.8	–	–	–
		SBalp	12 (men)	178.7 ± 8.7	77.4 ± 10.6	–	–	–
								–
Vernillo et al., 2017	Elite	SBalp	8 (2 women)	178.4 ± 9.8	78.1 ± 12.1	–	–	–

*Unless specified otherwise, data are reported as mean ± standard deviation. SBfs (freestyle), SBx (snowboard-cross), SBalp (alpine).*

### Aerobic Fitness

Maximum oxygen uptake (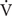
O_2max_) represents an accurate index of the integrated function of respiratory, cardiovascular, and muscular systems during exercise. Its importance for endurance performances is well and broadly established (Bassett and Howley, 2000). Within the literature, only two reports of aerobic fitness in elite snowboarders (**Table 1**) have been published thus far. The analysis of these reports showed 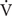
O_2max_ of ∼50 mLO_2_⋅kg^-1^⋅min^-1^ with a mean aerobic peak power output ranging from 3.5 to 5.3 W⋅kg^-1^. To the best of our knowledge, there are no other studies on aerobic characteristics of elite snowboarders. However, the importance of 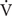
O_2max_ as a determinant factor of success in snowboarding has been called into question. This skepticism can be attributed to a study showing that 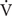
O_2max_ was unrelated to the performance level of snowboarders (Vernillo et al., 2016a). This conclusion contradicts the findings of Neumayr et al. (2003), who reported one of the crucial factors to determine success in professional alpine skiing was high levels of aerobic power. However, it must also be acknowledged that the importance of 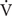
O_2max_ as a determining factor in alpine skiing has been similarly questioned (Maffiuletti et al., 2006) since 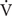
O_2max_ did not discriminate between skiers of different levels (Haymes and Dickinson, 1980; Brown and Wilkinson, 1983; White and Johnson, 1991; Impellizzeri et al., 2009). Given the singular study that found that 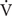
O_2max_ was not associated with success in snowboarding, further efforts are needed to confirm and corroborate such a conclusion. Yet even if this is confirmed, it remains unlikely that the aerobic system can be considered as a determinant factor for success in competitive snowboarding, as in alpine skiing. With regards to aerobic training, although there is no literature reviewing the influence of this specific component upon snowboarding performance, 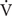
O_2max_ (and aerobic fitness in general) has been emphasized for its role in recovery (rather than energy provision) as for alpine skiing (Maffiuletti et al., 2006; Turnbull et al., 2009). Indeed, an efficient aerobic system is essential for recovery between competition runs, as well as to sustain the overall competition and on- and off-snow training season. For example, heart rate is a valid and reliable tool to monitor exercise intensity (Achten and Jeukendrup, 2003), even in snowboarding (Sporer et al., 2012). Accordingly, it has been used to determine the exercise intensity of training sessions. Kipp (1998) observed an average heart rate of 92% of predicted maximum heart rate during a halfpipe run in three elite American SBfs athletes. Arruza et al. (2005) used a manipulated training environment to examine the relationship between perceived fatigue and heart rate of five elite Spanish SBfs athletes. They reported that training demand was significantly related to heart rate (*r* = 0.74). Of note, on a daily on-snow training session SBalp athletes displayed high work loads relative to their individual fitness, maintaining a mean heart rate of ∼75–80% of the maximum heart rate (Vernillo et al., 2016a). In summary, the available data suggests that performance in snowboarding is not significantly determined by aerobic fitness, though the potential advantages of aerobic fitness for snowboarders in terms of training and recovery should be acknowledged.

### Muscular Strength and Power

Elite snowboarders present significant leg strength values, as shown in **Table 2**. Anecdotally, it seems that there are no strength differences among the different snowboarding disciplines. However, there has yet to be any investigation into the potential differences in trunk and upper limb muscular strength. As for alpine skiing (Hintermeister et al., 1995, 1997; Berg and Eiken, 1999), in snowboarding the load on the muscle system can be directly influenced by the accelerative force, relative to body weight, and the velocity of the snowboarder. Muscular strength and power in snowboarders have primarily been measured on the lower limb muscles, particularly the quadriceps. This is probably because injuries (especially those involving the knee) are common in elite snowboarding (Torjussen and Bahr, 2005; Bakken et al., 2011). Therefore, insufficient quadriceps muscle strength may limit the snowboarders’ ability to withstand the high forces and loads on the muscle system during snowboarding competitions, also increasing the risk of injuries. Additionally, preliminary evidence suggests a contribution from the lower leg muscles to the overall forces applied during snowboarding (Falda-Buscaiot and Hintzy, 2015). It seems then that possessing greater strength and endurance in the legs would be advantageous in snowboarding. Much of the current strength literature in snowboarding descriptively describes the general strength capacity of snowboarders. One exception comes from Gathercole et al.’s (2015) work with elite Canadian SBx athletes, where they investigate the feasibility of the counter movement jump test to examine the effect of both acute fatigue and training-induced adaptations. In general, little attention has been paid to the strength requirements of single- or multiple-day training/race. Therefore, studies clarifying the influence of strength training on the physiological response to snowboarding, and investigating the potential positive effects of strength training on snowboarding performance are necessary.

**Table 2:** Strength, power and jumping ability in snowboarders reported in the literature.

Study (year)	Competitive level	Discipline	Sample size	Isometric quadriceps force (N)	Leg press power (W⋅kg^-1^)	Jumping height (cm)		Jumping power (W⋅kg^-1^)		Jumping force (N⋅kg^-1^)	
			
						CMJ	SJ	CMJ	SJ	CMJ	SJ
Platzer et al., 2009	Elite	–	16 (women)	–	range: 4.46–6.54	range: 23.0–37.3	–	–	–	–	–
			21 (men)	–	range: 5.42–7.69	range: 32.5–48.9	–	–	–	–	–
											
Gathercole et al., 2015	Elite	SBx	5 (3 women)	–	–	45 ± 9	–	53.9 ± 5.5	–	20.7 ± 2.3	–
											
Vernillo et al., 2016a	Elite	SBx	10 (men)	680.1 ± 76.8	–	–	–	71.6 ± 3.1	68.5 ± 7.4	–	–
		SBalp	10 (men)	731.9 ± 181.9	–	–	–	73.0 ± 3.7	70.6 ± 7.3	–	–
											
Vernillo et al., 2016b	Elite	SBfs	10 (men)	684.6 ± 137.2	–	–	–	–	–	26.8 ± 2.8	–
		SBx	11 (men)	674.1 ± 78.8	–	–	–	–	–	26.2 ± 2.8	–
		SBalp	12 (men)	754.6 ± 162.1	–	–	–	–	–	27.1 ± 3.4	–

*Unless specified otherwise, data are reported as mean ± standard deviation. SBfs (freestyle), SBx (snowboard-cross), SBalp (alpine).*

### Strength Asymmetry

**FIGURE 1:**
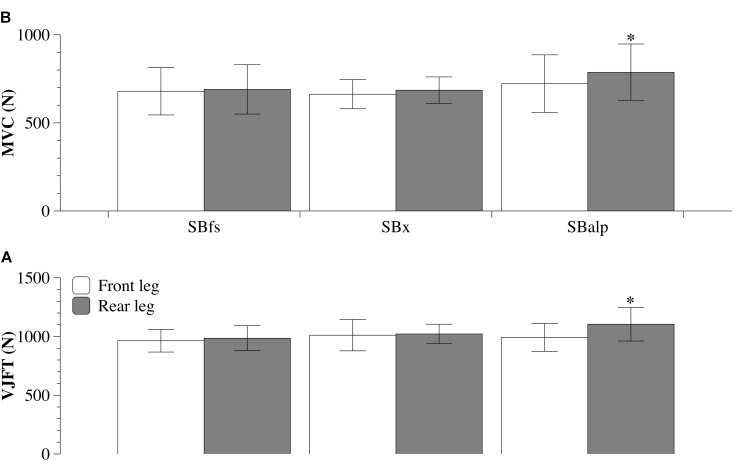
Mean ± standard deviation of the results from the isometric maximal voluntary contraction test (MVC, **A**) and the vertical jump force test (VJFT, **B**) in elite Italian snowboarders. Based on data from Vernillo et al. (2016b). ^∗^Significantly different from the front leg (*P* < 0.001). SBfs (freestyle), SBx (snowboard-cross), SBalp (alpine).

Strength asymmetry refers to the relative difference between legs in maximal force capacity. Its quantification can be useful in identifying athletes at increased risk of incurring lower-limb musculoskeletal injuries (Impellizzeri et al., 2007). Due to an asymmetrical position on the board [with the left or right leg in front (regular or goofy position, respectively)], snowboarders can be at risk of developing strength asymmetry between the two legs. We have recently published data tested this hypothesis (**Figure 1**) (Vernillo et al., 2016b), where the strength asymmetry of 33 elite snowboarders [SBfs (*n* = 10), SBx (*n* = 11) and SBalp (*n* = 12)] was assessed. All athletes underwent tests with the same protocol, consisting of an isometric maximal voluntary contraction of both the front and rear leg, and a vertical jump test on a portable force platform [with asymmetry measured by a parallel wooden platform leveled with the force platform (Impellizzeri et al., 2007)]. Only SBalp athletes presented a ∼10.5% strength asymmetry, favoring the rear leg (**Figure 1**). This likely occurs due to greater weight distribution on the rear leg during snowboarding that could reflect an increased adaptation of muscle characteristics, such as size and volume. We confirmed this hypothesis observing a ∼14% difference in muscle architecture between the front and the rear leg (i.e., a lower pennation angle associated with a greater fascicle length), which also suggests the presence of a morphological asymmetry in elite SBalp athletes (Vernillo et al., 2017). In summary, it appears that functional and morphological asymmetries are only present in SBalp. A cut-off of ± 15% is commonly accepted as being clinically relevant in relation to developing a potential harmful strength asymmetry (Impellizzeri et al., 2007; Schmitt et al., 2012). However, whether such asymmetry represents a potential risk factor for injury, or an intrinsic characteristic remains a point of ongoing debate. This is because strength asymmetry can also be considered a peculiarity of many sports due to a constant training overload on the dominant limb. Therefore, strength asymmetry in snowboarders should be taken into consideration in terms of predisposition to lower limbs musculoskeletal injuries. It should further be acknowledged as practically relevant on the assessment of functional muscular deficits consequent to injury as well as exercise prescription. Given the beneficial (but limited) data acquired thus far, further studies that include athletes of more varying attributes and backgrounds within snowboarding could potentially yield more generalized results.

## The Relationship Between Physiological Tests and Snowboarding Performance

Snowboarding is comprised of various disciplines that may differ in their physiological and technical requirements. Accordingly, success in snowboarding may be related to a complex interaction of multiple variables. For example, SBx and SBalp performances are positively related both to body dimension and relative body composition (Vernillo et al., 2016a). However, it should be stressed that (to date) only the above-mentioned study has sought to measure anthropometric variables and correlate them with indices of snowboarding performance. This was largely descriptive and done with relatively small sample sizes. Thus far, no snowboarding specific training interventions (or comparison with non-snowboarding control groups) have been conducted. In addition, more specific physical characteristics of snowboarders (such as the somatotype distribution) have not yet been tested. Therefore, the relation between percentage body fat and other measures of body dimensions with snowboarding performance is yet to be clearly established. Recent data on elite men Italian snowboarders showed that 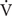
O_2max_ (as well as the ventilatory thresholds) was not correlated with performance (Vernillo et al., 2016a). In contrast, absolute and relative power output assessed during incremental cycling tests seem to be more important in determining snowboarding performance. As reported by Platzer et al. (2009), peak power output (normalized for body mass) was strongly related to success among women snowboarders (*r* = 0.85). This observation was confirmed and extended by Vernillo et al. (2016a), reporting that absolute and normalized power outputs (as well as power output at the first and second ventilatory threshold) were strongly related to men SBx and SBalp performances (*r* = -0.84 to -0.93). To date, these are the only studies that have sought to compare snowboarding performance with aerobic-measured cycle ergometer values. Taken together, these results highlight that power output seems to represent a greater indicative value of the snowboarding performance than do ventilatory responses. This suggests that muscle power is a better predictor of snowboarding performance than, for example, 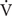
O_2max_. Finally, early research found muscular power (measured by isokinetic leg press) to be strongly correlated to snowboarding superiority among Austrian snowboarding team members (Platzer et al., 2009). Vernillo et al. (2016a) confirmed and extended the previous observations, reporting isometric muscle strength at the quadriceps to be strongly related to men SBx and SBalp performances (*r* = -0.93 to -0.97). Therefore, muscle strength and power are important determinants in snowboarding competition. Indeed, high values of these parameters may allow the snowboarders to train/compete at a reduced percentage of their maximal strength/power, thereby reducing the metabolic consequences of sustained muscular activities, and to withstand the high forces of snowboarding. A strong association has also been found between leg stiffness [measured form flight and contact times during multi-rebound jumps (Dalleau et al., 2004)] and men SBx and SBalp performances (*r* = -0.85 to -0.89) (Vernillo et al., 2016a), highlighting the important role of muscular stiffness regulation to maintain the muscle force generating capacity. Of interest, the ability to generate explosive strength (e.g., peak power determined during counter movement jumps) was not significantly correlated to indexes of snowboarding performance (Platzer et al., 2009; Vernillo et al., 2016a), displaying that snowboarding, as alpine skiing, should not be considered an “explosive” sport. Despite this conclusion, the functional significance of counter movement jump must be acknowledged. For example, while studying elite Canadian SBx athletes, Gathercole et al. (2015) observed counter movement jumps to be a suitable monitoring tool for the detection of both acute fatigue and training-induced adaptations. Factors such as technical ability as well as trunk and upper body muscle strength might also influence the athletes’ capacity to withstand the forces generated during snowboarding, and further serve to generate speed. Such components may prove invaluable when combine with the physiological assessment of snowboarders in the pursuit of future studies.

### Determinants of Snowboarding Performance

In the study of 37 elite Austrian snowboarders (21 men; 16 women), Platzer et al. (2009) address the key determinants of snowboarding performance. Variables for each participant included anthropometric measures (height and body mass) as well as physiological and functional markers (aerobic peak power output, leg and core power, stability index, bench press/pull strength and maximum push off speed on an indoor start simulator). Using a multiple regression analysis, it was shown that the variables considered explained 61 and 73% of the variance of WC points in women and men, respectively. Examining the different disciplines, the same variables explained 61 and 98% of the variance of FIS points in women’s SBalp and SBx events, respectively, as well as 78% of variance of FIS points in men’s SBfs halfpipe events. It was concluded that anthropometric, physiological and functional variables are a good predictor for snowboard performance in women but not for men, arguing that other performance-determining variables (e.g., psychological, equipment and coordination) might play a role on influencing the snowboard performance.

## Conclusion and Future Research Directions

Contemporary snowboarding literature describes the physiological and performance characteristics of snowboarders. As we have highlighted throughout this preliminary review, important gaps in our physiological and performance knowledge of snowboarding still exist. The determinants of snowboarding performance remain unclear, but may be attributed to a variety of trainable variables. Improvement of performance analysis within snowboarding is therefore required to better understand the internal and external loads experienced by this population of athletes. Without implementing such methods, practitioners lack a specific understanding of the sport regarding the workloads, durations and stress involved. Therefore, they would be limited to speculate on training requirements for these athletes. Ideal training regimens to optimize physiological markers and snowboarding performance have not yet been identified. Accordingly, well-designed training studies are needed to confirm the indications presented in this preliminary review. Quantifications of physiological and physical requirements of one (or several days) of training and races are needed in order to optimize athletic performance. There remains a need to provide snowboarders’ physiological and physical profiles that are not biased by a nation-specific training methodology. An increase in research of snowboarding could positively benefit the overall level of professionalism within the sport. Until further research is conducted, snowboarding provides an ongoing challenge for exercise physiologists, sport scientists, and conditioning experts alike in building accurate and structured training regimes. We hope this preliminary review will promote further research in all the aspects highlighted in it.

## Author Contributions

GV, CP, and GT conceived and designed the research, analyzed the data, prepared the figure, drafted the manuscript, edited and revised the manuscript, and approved the final version of the manuscript.

## Conflict of Interest Statement

The authors declare that the research was conducted in the absence of any commercial or financial relationships that could be construed as a potential conflict of interest. The reviewer KM and handling Editor declared their shared affiliation.
